# Inflammatory bowel disease and patterns of volatile organic compounds in the exhaled breath of children: A case-control study using Ion Molecule Reaction-Mass Spectrometry

**DOI:** 10.1371/journal.pone.0184118

**Published:** 2017-08-31

**Authors:** Lorenzo Monasta, Chiara Pierobon, Andrea Princivalle, Stefano Martelossi, Annalisa Marcuzzi, Francesco Pasini, Luigi Perbellini

**Affiliations:** 1 Institute for Maternal and Child Health – IRCCS “Burlo Garofolo”, Trieste, Italy; 2 Occupational Medicine, Department of Public Health and Community Medicine, University of Verona, Verona, Italy; Yale University Yale School of Public Health, UNITED STATES

## Abstract

Inflammatory bowel diseases (IBD) profoundly affect quality of life and have been gradually increasing in incidence, prevalence and severity in many areas of the world, and in children in particular. Patients with suspected IBD require careful history and clinical examination, while definitive diagnosis relies on endoscopic and histological findings. The aim of the present study was to investigate whether the alveolar air of pediatric patients with IBD presents a specific volatile organic compounds’ (VOCs) pattern when compared to controls. Patients 10–17 years of age, were divided into four groups: Crohn’s disease (CD), ulcerative colitis (UC), controls with gastrointestinal symptomatology, and surgical controls with no evidence of gastrointestinal problems. Alveolar breath was analyzed by ion molecule reaction mass spectrometry. Four models were built starting from 81 molecules plus the age of subjects as independent variables, adopting a penalizing LASSO logistic regression approach: 1) IBDs vs. controls, finally based on 18 VOCs plus age (sensitivity = 95%, specificity = 69%, AUC = 0.925); 2) CD vs. UC, finally based on 13 VOCs plus age (sensitivity = 94%, specificity = 76%, AUC = 0.934); 3) IBDs vs. gastroenterological controls, finally based on 15 VOCs plus age (sensitivity = 94%, specificity = 65%, AUC = 0.918); 4) IBDs vs. controls, built starting from the 21 directly or indirectly calibrated molecules only, and finally based on 12 VOCs plus age (sensitivity = 94%, specificity = 71%, AUC = 0.888). The molecules identified by the models were carefully studied in relation to the concerned outcomes. This study, with the creation of models based on VOCs profiles, precise instrumentation and advanced statistical methods, can contribute to the development of new non–invasive, fast and relatively inexpensive diagnostic tools, with high sensitivity and specificity. It also represents a crucial step towards gaining further insights on the etiology of IBD through the analysis of specific molecules which are the expression of the particular metabolism that characterizes these patients.

## Introduction

Inflammatory bowel diseases (IBDs), which comprise Crohn’s disease (CD) and Ulcerative Colitis (UC), are chronic inflammatory conditions of the gastrointestinal tract that profoundly affect the quality of life and have been gradually increasing in incidence, prevalence and severity in many areas of the world. [1–5] Around 25% to 30% of all diagnoses are made in the first two decades of life. [1,6] Among childhood onset-IBD, there is an especially rising incidence of CD that is approximately 3/100,000. [3] The prevalence in the pediatric population (< 20 years of age) is reported to be 58/100,000 for CD and 34/100,000 for UC. [4]

Failure to diagnose and induce disease remission during the peri-pubertal period can have significant consequences such as missed pubertal growth spurt and reduced adult height, [7] or low bone mineral density leading to an increased long-term risk of fractures. [8]

Patients with suspected IBD require a careful history and clinical examination along with blood tests. However, normal laboratory investigations cannot exclude a diagnosis of IBD. [6,9] Definitive diagnosis relies on endoscopic and histological findings. [9] Gastrointestinal endoscopy and colonoscopy should be undertaken in any patient with suspected IBD. [9] Multiple mucosal biopsies should be obtained for histopathological examination. [6] Other ways of investigating the small bowel in CD are capsule endoscopy and magnetic resonance imaging. They can provide details about the extent of inflammatory changes in the mucosa and are also able to identify smaller superficial mucosal lesions without radiation. [2] The only non-invasive high sensitivity (73.5–100%) marker for gut inflammation is fecal calprotectin, which has, however, low specificity (65.9–97.9%). [10]

No simple, fast and cheap test for diagnosing and monitoring intestinal inflammation in IBD is available at present.

There is strong evidence to suggest that particular disorders that increase oxidative stress can be detected by molecular analysis of exhaled air. [11] Breath analysis represents a new diagnostic technique that started in the 1970s when Pauling et al. detected approximately 250 components in human breath using gas chromatography. [12] Various analytical techniques have been used to detect exhaled VOCs: the most commonly used are mass spectrometry (MS)-based techniques, [11] among which the leading is gas chromatography (GC-MS), which are followed by the use of nanoparticles sensor arrays. [13] Several studies have shown that VOCs profile can be helpful to diagnose several diseases, [14] including lung cancer, [15,16] breast cancer, [17,18] diabetes mellitus, [19] hepatic cirrhosis, [20] active tuberculosis, [21] cystic fibrosis [22] and preeclampsia. [23]

Metabolic derangement in IBDs was initially studied using the headspace of feces and urine. Probert compared the VOCs profile in the headspace gas emitted from fecal samples from IBD patients, healthy subjects and patients with infectious diarrhea. He found a specific pattern of compounds strongly associated with the alteration of intestinal homeostasis. [24] Another study demonstrated the potential application of fecal VOC analysis in diagnosing IBD in a pediatric cohort. [25] The headspace of urine in IBD patients showed a different VOC profile, with the suggestion that altered gut permeability is reflected in urinary profiles. [26]

A recent review investigated the role of VOCs breath analysis in the diagnosis of gastrointestinal diseases, including IBDs. [27] Lipid peroxidation appears to be the main mechanisms behind the changes in the VOCs profile in both CD and UC patients. Pentane, ethane, propane and isoprene appear to present consistently higher levels in patients with IBD compared to controls. Also fractional exhaled nitric oxide (FENO) measurements in patients with Crohn's disease has been investigated as a marker of active inflammation. Significantly higher levels of FENO were observed in CD patients with clinically active disease compared to CD patients in clinical remission. [28]

Hicks et al. has shown that exhaled breath VOCs profiling can distinguish IBDs adult patients from healthy controls. [29] VOCs belonging to the aldehyde group (butanal and nonanal) are elevated in both UC and CD, and are, especially in the latter, a marker of oxidative stress. Also volatile sulfur-containing compounds (dimethyl sulfide and hydrogen sulfide) were shown to be able to distinguish CD patients from UC and controls. Hydrogen sulfide was significantly lower in CD, while ammonia was significantly lower in UC compared to healthy controls. [29]

Only one study verified the presence of a specific VOCs pattern in the alveolar air of children with IBD, [30] and found that the values of three specific VOCs (1-octene, 1-decene, E-2-nonene) could discriminate between IBD and controls. However, no distinctive pattern could be identified for CD and UC.

The primary aim of our study is to investigate whether pediatric patients with IBD have specific VOCs patterns when compared to control subjects. Patients will be divided into four groups: CD, UC, controls with gastrointestinal symptomatology, and surgical controls with no evidence of gastrointestinal problems. Having identified specific VOCs patterns, the second aim of the study was to try to understand how discriminating molecules could be linked to the IBDs.

## Methods

### Cases and controls

The study was approved (RC 1/12) by the Technical Scientific Committee of the Institute for Maternal and Child Health—IRCCS “Burlo Garofolo” of Trieste, Italy. All enrolled patients and/or their parents or caregivers signed an informed consent form prior to their enrollment.

From June 2012 to June 2013, we enrolled patients aged 10–17 years affected by IBD (both ulcerative colitis and Crohn’s disease) (“cases”), other gastrointestinal diseases (“gastro controls”) and subjects without gastrointestinal problems (“healthy controls”). Diagnoses of ulcerative colitis and Crohn’s disease were made according to the ESPGHAN and NASPGHAN guidelines for the pediatric population. [31] All cases and gastro controls were enrolled at the outpatients service of the Gastroenterology Unit of the Institute for Maternal and Child Health—IRCCS Burlo Garofolo of Trieste, Italy. Subjects without gastroenterological problems were enrolled at the Day Surgery among patients hospitalized for issues not related to gastroenterology (orthopedic, otolaryngology, eye, dental, urology surgery): these patients were all carefully evaluated to exclude those with gastrointestinal symptoms. At the time of air sampling, which was carried out in the morning, all subjects has been fasting at least since midnight. Breath sampling in all day surgery controls was done pre-operatively. Additional information on their medical history and ongoing therapies was collected. Patients with IBD were also evaluated using the Pediatric Ulcerative Colitis Activity Index (PUCAI) [32], the Pediatric Crohn’s Disease Activity Index (PCDAI) [33]. Both indexes are reported in Tables A and B of S1 Text. The Paris disease classification has been used to classify IBD cases for localization and to capture the dynamic features of the disease phenotype. [34]

### Alveolar air sampling

**Fig 1:**
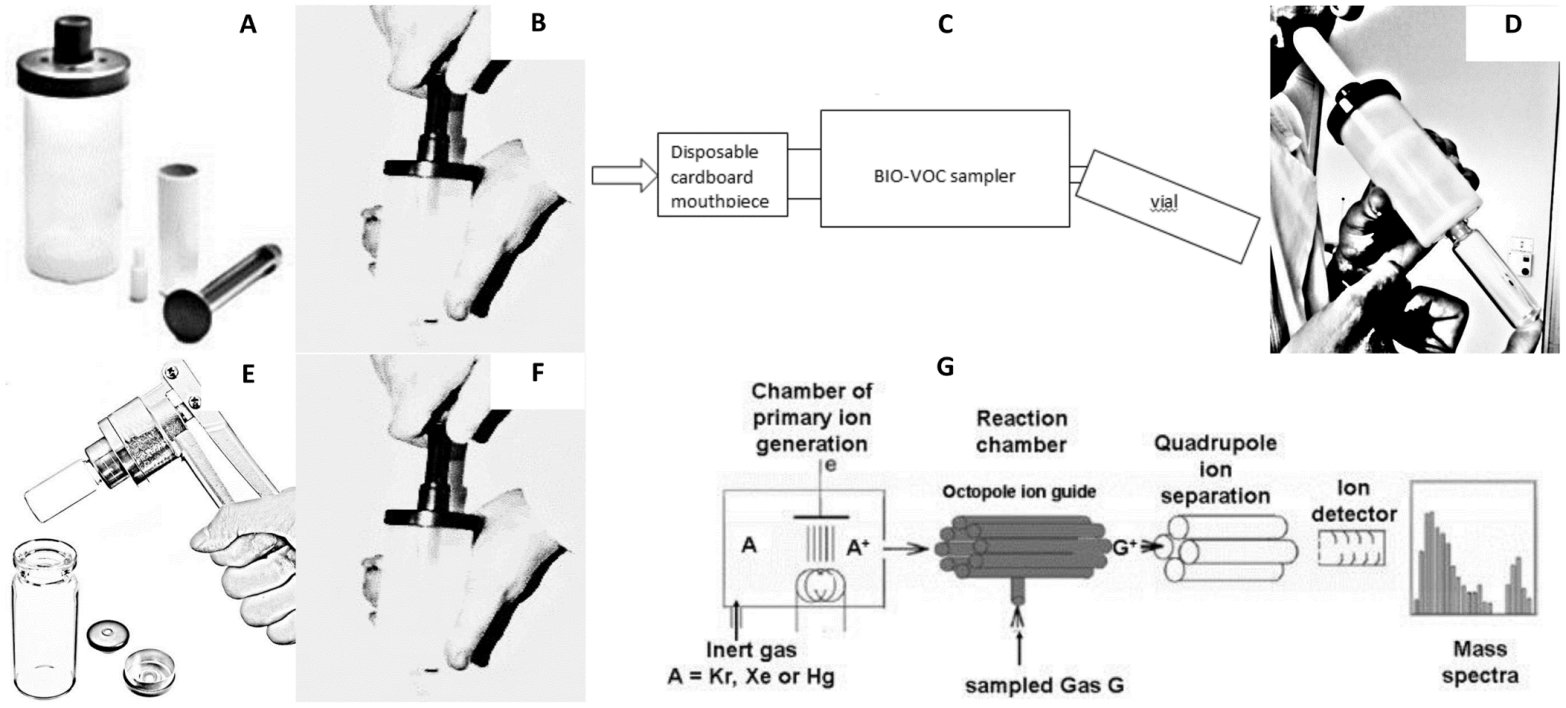
Explanation of the sampling procedure and the VOCs measurement by IMR-MS. A) breath sampler with disposable cardboard mouthpiece and the pushrod; B) connect the pushrod to the sampler and flush the sampler by pulling and pushing the rod in and out two or three times; C) remove to rod and connect the disposable mouthpiece to the sampler, placing the glass vial on the other side; D) have the patient breath normally and then keep exhaling trough the mouthpiece until their lungs are emptied; E) crimp airtight the glass vial with the appropriate crimp cap; F) throw away disposable mouthpiece and clean the breath sampler by flushing it two/three times using the pushrod; G) the glass vial, preserved at –20°C, is analyzed with the IMR-MS method, schematized here (reported from Defoort and colleagues [35]) and described in detail in Hornuss and colleagues [36].

For breath sampling, subjects were asked to exhale once through a device called Bio–VOC^™^ breath sampler (Markes International Ltd., Llantrisant, UK) into a 20 ml volume glass vial (the Bio–VOC^™^ sampler avoids rebreathing). All glass vials had been previously sterilized and sealed individually. After completing exhalation, the glass vial was crimped airtight with the appropriate crimp cap. Two samples of expired air were collected for each subject to increase the possibility of obtaining at least one properly sealed sample. In addition, a glass vial was sealed with environmental air present at the same time and in the same place as each exhaled air sample. Vials were preserved at –20°C up to the moment of the Mass Spectrometry (MS) analysis. Fig 1 shows the steps of the sampling procedure and VOCs analysis.

### Equipment

VOCs in alveolar breath and in environmental samples were analyzed using the “Airsense” Ion Molecule Reaction–Mass Spectrometry (IMR–MS) from V&F (medical development GmbH, Absam, Austria). The soft ionization process was performed via ion beams interacting with the gas sample, as already reported by Hormuss and colleagues. [36] The vials were placed in a V&F autosampler, heated up to 65°C and dynamically transferred to the V&F Airsense. The spectrometer measures the concentration of products in a sample. These products mainly represent molecules existing in traces in the sample but may, in some cases, also represent fragments of other molecules generated by the soft ionization occurring in the instrument.

The concentration of 97 volatile compounds (masses from 16 to 123) was measured in all samples. Thirty-one compounds had a known chemical structure (directly or indirectly calibrated with calibration gasses), while 66 groups of products were known only for their molecular weight (MW). A direct calibration was carried out for 23 chemical compounds: Acetylene, Ethane, Formaldehyde, Methanol, Acetonitrile (ACN), Formic Acid, Acetic Acid, Ethylene, Propene, Acetaldehyde, Butadiene, Butanol, Methyl Ethyl Ketone (MEK), Acetone, n–Propanol, Isoprene, n–Pentane, Benzene, n–Hexane, Toluene, n–Heptane, CO_2_ and O_2_. Our aim was to calibrate a panel of compounds between 16 and 123 Dalton, including petroleum-related products, micropollutants measured by various authors, molecules derived from human or animal metabolism (acetone, isoprene, n-pentane), molecules that are present in foods or their metabolites (acetic acid, formic acid, formaldehyde, methanol), and products found in alveolar air by various authors and that could have useful biological meanings. These products provided indications on the quality of the results obtained.

CO_2_ and O_2_ were measured by a specific detector with variation coefficients lower than 1%. For the reported compounds, the reproducibility of the assays was assessed by analyzing 30 environmental air samples collected in the same room, in six replicates on five different days over a three weeks period. The intra-assay (comparison of samples collected on the same day) coefficients of variation were less than 10% for Ethane, Formaldehyde, Methanol, ACN, Formic Acid, Ethylene, n-Butanol and n–Pentane. For the other products the intra-assay coefficients of variation were less than 20%, except for Acetone (24%) and Acetic Acid (35%). The inter-assay (comparison of samples collected on different days) coefficients of variation were lower than 10% for Formaldehyde, Methanol, ACN, Formic Acid and n-Butanol, and lower than 20% for the other products. The inter-assay coefficients of variation were 27% for Acetone and Ethane, and 40% for Acetic Acid. Coefficients of variation of ppb concentrations are considered to be highly satisfactory if below 20%. The values above 20% we obtained can be considered as acceptable.

Benzene was used for the indirect calibration of other eight molecules: Methane, HNO_2_, N_2_O, NO, H_2_S, H_2_O, Ammonia (NH_3_), Sulfur Dioxide (SO_2_). This model represents a semi–quantitative calibration procedure which is commonly used in (multicomponent) analytical devices.

The measured VOCs are given as absolute concentrations (ppm) and as volume percent for CO_2_ and O_2_, these latter gases being used to provide information on the quality of environmental or alveolar samples. CO_2_ values lower than 2% in alveolar air samples were presumed to be associated to missampling or to inadequate vial crimping: these samples were excluded.

### Statistical analyses

We first described the sample of controls and patients with Crohn’s disease (CD) and with ulcerative colitis (UC). We then graphically represented, as Δ between medians of exhaled and environmental air, the comparison between the VOCs profile of all breath samples and that of all the environmental samples. We also graphically compared breath samples of CD and UC patients with controls values, again as Δ between breath samples of CD or UC patients and controls.

In order to establish if the VOCs were to be considered endogenous or exogenous, we ran a t–test for each of the 98 compounds, and verified whether the values in the environmental air samples were significantly higher than in the exhaled breath samples. If so, the “exogenous” compound was excluded from the regression models exposed below. Compounds with higher concentrations in the environment if compared to the exhaled breath have a partial pressure inducing pulmonary absorption, and their alveolar concentrations will be in constant equilibrium with the ones in the environment. Consequently they will substantially not be informative on the physiopathological conditions of the organism. The choice of focusing on compounds with alveolar concentrations higher than environmental ones was meant to restrict the panel to products primarily associated with specific metabolic (physiological or pathological) conditions, avoiding interferences attributable to “environmental pollutants”. The exclusion was also justified by the fact that in the environmental air samples few of these exogenous molecules had significantly different values in cases and controls, probably due to environmental differences in the outpatient clinic in which samples from the two groups were taken.

For the elaboration of a predictive model that might allow for future generation of a diagnostic tool, and considering the large number of independent variables involved in the analysis, we decided to adopt a Lasso (Least Absolute Shrinkage and Selection Operator) logistic regression (LLR) approach. [37,38] By shrinking the estimates of the regression coefficients towards zero relative to the maximum likelihood estimates, this penalizing estimation method prevents any overfitting that may arise as a consequence of either collinearity or high–dimensionality of independent variables. This method allows to shrink the regression coefficients adopting a tuning parameter λ, which controls the amount of shrinkage that is applied to the estimates. In addition, the shrinkage of some coefficients to zero reduces the number of covariates in the final model, allowing us to avoid using classical stepwise regression methods, which are strongly criticized for their lack of consistency. [39] Independent variables (molecules) were standardized to allow for optimal penalization.

In particular, we adopted an iterated LLR approach. [40] First, we used a 50–fold cross–validated LLR to reduce the number of variables in the model, eliminating all variables if coefficients were 0. Then we used this set of variables in a two–step iterated 50–fold cross–validated LLR, [40] in which the first LLR generated penalized weights to be used in a second adaptive LLR. [41]

LLR was used to generate four different models with the VOCs remaining after the exclusion of the exogenous ones, plus age as independent variables, diverging in terms of dependent variable: 1) IBD patients vs. controls (gastroenterological and healthy); 2) IBDs vs. gastroenterological controls; 3) Crohn’s disease (CD) patients vs. patients with ulcerative colitis (UC); 4) the first model was then replicated using only the molecules that had been directly or indirectly calibrated, with the intent of generating a model with unambiguously identified molecules. The intent of the first model is to try and separate IBDs from a “real population” mix, made of children with and without gastrointestinal problems. The second model aims at reproducing the situation that is found in a gastroenterology outpatient clinic. The third model represent a second step in diagnosis, moving from the identification of IBD to the separation between CD and UC, while also addressing the question of the differences in VOCs profiles between CD and UC patients. The fourth model has been deprived of the unknown molecules. This represents a limitation compared to the first model, but, being based on known molecules only, this latter model can be replicated more easily.

What varies from model to model is the way in which the λ value was selected in the first LASSO. In some cases, even when we had the possibility of selecting an optimal λ, based on the graph representing the penalization of the variables involved, we chose to adopt a less penalizing λ, obtaining as a result a larger number of variables to be included in the iterated LASSO procedure.

Analyses were carried out with Stata/IC 11.2 (StataCorp LP, College Station, TX, USA) and with R version 2.15.1 (The R Foundation for Statistical Computing, Vienna, Austria), and “penalized” (Goeman JJ. Penalized R package, version 0.9–42) and “polywog” (Kenkel B, Signorino CS. Bootstrapped Basis Regression with Oracle Model Selection, version 0.2–0) R packages.

## Results

A total of 234 subjects was enrolled in the study over a one year period: 67 cases (33 UC and 34 CD patients), and 167 controls (65 gastrointestinal controls and 102 healthy controls) (Table 1). After receiving quick and simple instructions, all subjects carried out the air sampling without any difficulty. Cases and gastroenterological controls are described in Tables A to C in S2 Text.

**Table 1:** Description of the sample of inflammatory bowel disease cases and controls enrolled in the study (children 10 to 17 years of age).

	UC (33)	CD (34)	Gastro Ctrls (65)	Healthy Ctrls (102)
Sex	F 15; M 18	F 16; M 18	F 27; M 38	F 45; M 57
Age	14 (12–16)	15 (14–16)	12 (11–15)	13 (11–14)

F: Females; M: Males; UC: Ulcerative colitis; CD: Crohn’s disease; Gastro Ctrls: gastroenterological controls; Healthy Ctrls: Healthy controls. Age is expressed in years as median and interquartile range in parenthesis.

First, we analyzed the differences in concentration of the 97 molecules present in the environment and in the alveolar air of cases and controls, and excluded from further analyses 13 molecules with significantly lower values in the alveolar air compared to environmental air (M27, Ethane, Formaldehyde, Methanol, Formic Acid, SO_2_, NO, H_2_S, M31, M32, M48, M49, M80), which were thus defined as exogenous. Some of the excluded compounds, such as hydrogen-sulfide, could also have an endogenous nature. As explained above, however, the significantly higher presence of such compounds in environmental air if compared to exhaled breath would mean that most of the expired component would not be endogenous, and would thus be difficult to interpret.

**Fig 2:**
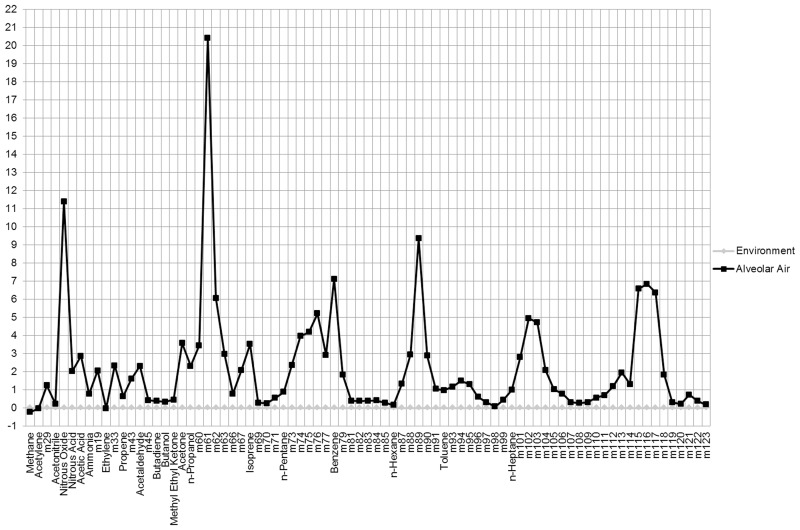
For the 81 molecules considered, difference between median values of alveolar and environmental air, with values standardized to environmental air.

**Fig 3:**
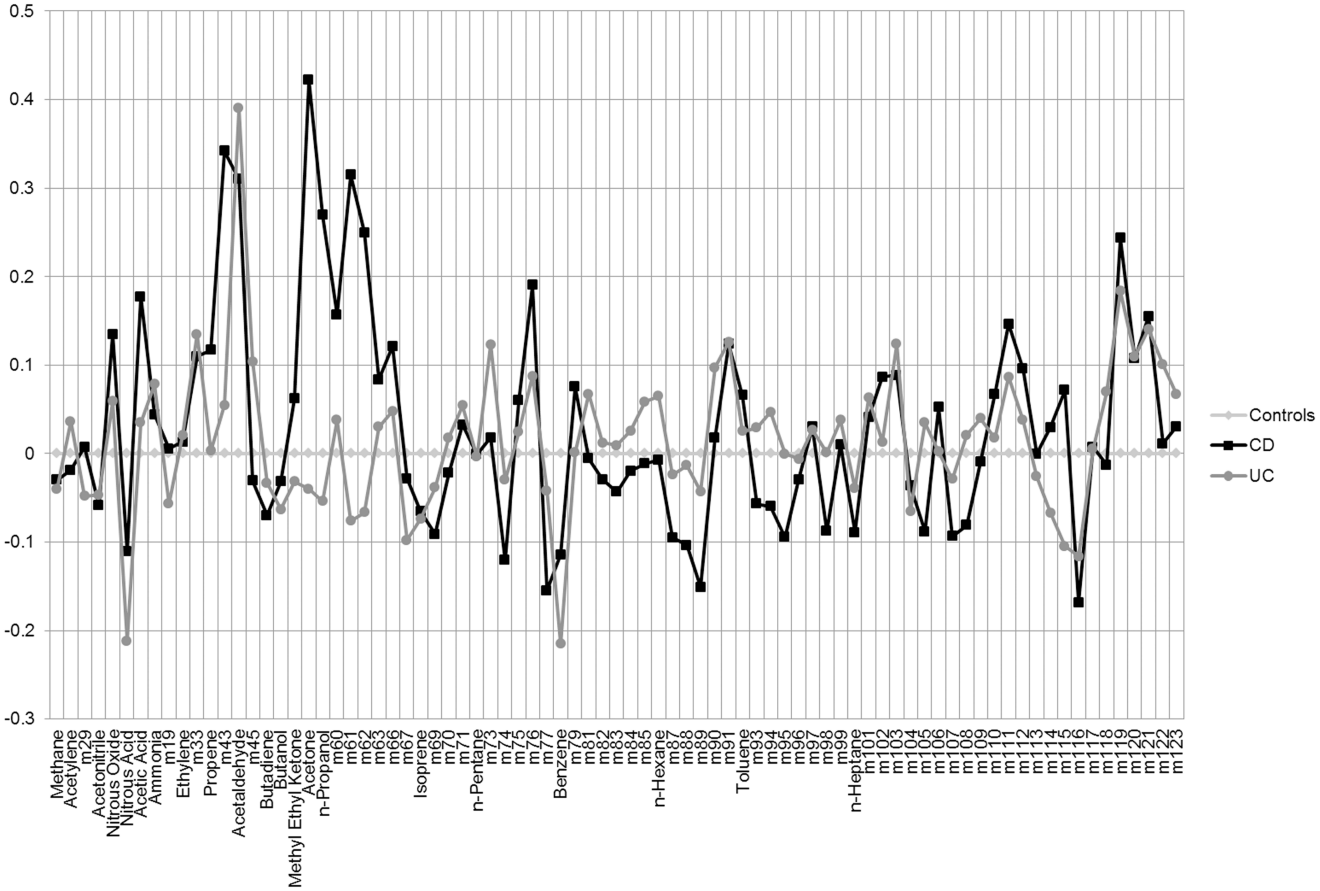
For the 81 molecules considered, VOCs profiles of median values of CD and UC patients compared to control subjects, with values standardized to the median values of control subjects.

Data on H_2_O, O_2_ and CO_2_ concentrations were employed to assess whether the samples had been collected properly, but were excluded from the models because of their particularly cumbersome presence. We thus remained with the 81 molecules (Table 2) listed in Fig 2, which shows the difference between median values of alveolar and environmental air, with values standardized to environmental air. Fig 3 also shows the VOCs profiles of median values of CD and UC patients compared with control subjects, with values standardized to the median of control subjects. Mean values and 95% confidence intervals for these molecules, as identified in environmental air samples and in exhaled breath samples of cases and controls, are reported in S1 Table.

**Table 2:** Volatile organic compounds measured by ion-molecule reaction-mass spectrometry, with indications on whether molecules were directly or indirectly calibrated, and which molecules were included in the regression models after comparison between environmental air and exhaled air samples.

Measured molecules (97)	Directly calibrated molecules (23)	Indirectly calibrated molecules* (8)	Molecules included in models (81)
CH_4_—Methane		x	x
C_2_H_2_—Acetylene	x		x
M27			
M29			x
C_2_H_6_—Ethane	x		
CH_2_O—Formaldehyde	x		
CH_4_O—Methanol	x		
C_2_H_3_N—Acetonitrile	x		x
N_2_O—Nitrous Oxide		x	x
CH_2_O_2_—Formic Acid	x		
HNO_2_—Nitrous Acid		x	x
SO_2_—Sulfur Dioxide		x	
H_2_O—Water		x	
O_2_—Oxygen	x		
CO_2_—Carbon Dioxide	x		
NH_3_—Ammonia		x	x
M19			x
C_2_H_4_—Ethylene	x		x
NO–Nitric Oxide		x	
M31			
M32			
M33			x
H_2_S—Hydrogen Sulfide		x	
C_3_H_6_—Propene	x		x
M43			x
C_2_H_4_O—Acetaldehyde	x		x
M45			x
M48			
M49			
C_4_H_6_—Butadiene	x		x
C_4_H_10_O—Butanol	x		x
C_4_H_8_O—Methyl Ethyl Ketone	x		x
C_3_H_6_O—Acetone	x		x
C_3_H_8_O—n-Propanol	x		x
C_2_H_4_O_2_—Acetic Acid	x		x
M60			x
M61			x
M62			x
M63			x
M66			x
M67			x
C_5_H_8_—Isoprene	x		x
M69			x
M70			x
M71			x
C_5_H_12_—n-Pentane	x		x
M73			x
M74			x
M75			x
M76			x
M77			x
C_6_H_6_—Benzene	x		x
M79			x
M80			
M81			x
M82			x
M83			x
M84			x
M85			x
C_6_H_14_—n-Hexane	x		x
M87			x
M88			x
M89			x
M90			x
M91			x
C_7_H_8_—Toluene	x		x
M93			x
M94			x
M95			x
M96			x
M97			x
M98			x
M99			x
C_7_H_16_—n-Heptane	x		x
M101			x
M102			x
M103			x
M104			x
M105			x
M106			x
M107			x
M108			x
M109			x
M110			x
M111			x
M112			x
M113			x
M114			x
M115			x
M116			x
M117			x
M118			x
M119			x
M120			x
M121			x
M122			x
M123			x

* Calibrated through Benzene

The 81 molecules plus the age of subjects were considered as independent variables in all four models. The results of the models are reported below.

### IBD (CD + UC) vs. controls (surgical + gastroenterological)

The first model is based on the comparison between all IBDs without distinction and all controls. The final resulting model comprises 18 VOCs plus age in years, and has the following formula:

- predicted probability = 1/(1+exp(– (–1.7610965 +0.4349465*Age –2.1312841*Methane –0.2097137*NitrousAcid +0.0011512*AceticAcid +0.0004002*Ammonia +0.0058942*Propene +0.0008888*Acetaldehyde –0.0232373*MethylEthylKetone –0.1222845*M69–0.0266032*M74 +0.1684388*M76–0.0489416*M79 +0.0425079*M81–0.0280041*M89 +0.0793372*M99–0.1427593*M105–0.0132398*M107–0.0539797*M115 +0.1597398*M118)))

Confidence intervals and standard error of the coefficients are reported in Table 3. The Area under the ROC curve (AUC) was 0.925 (95%CI: 0.889–0.961) (S1 Fig) and the performance of the model for sensitivity levels above 90% are reported in Table 4. The model could detect 96% of all IBD cases with a specificity of 69% (65% in gastro controls and 73% in surgical controls). In this case, 23 out of 65 gastro controls result as being false positives. No significant differences in the distribution of the diseases was found among these (Table 5). However, when the diseases are classified based on the presence (or plausibility) of an ongoing inflammatory process, while among the correctly classified two are found to be biliary duct atresias and one was a choledochal cyst, the false positives include a Behçet’s disease, a chronic intestinal pseudo-obstruction with ileostomy, and intestinal atresia, an infective ileitis, and a graft-versus-host disease.

**Table 3:** Variables and coefficients of the logistic regression model with outcome variables: Patients with inflammatory bowel disease vs. controls (surgical + gastroenterological).

Variables	Coefficient	Std. Error	95% CI
(Intercept)	-1.7610965	3.5213722	-8.030–2.522
Age (years)	0.4349465	0.1115950	0.408–0.718
Methane	-2.1312841	1.0351154	-4.340 –-1.422
Nitrous Acid	-0.2097137	0.1800298	-0.489–0.000
Acetic Acid	0.0011512	0.0007470	0.000–0.002
Ammonia	0.0004002	0.0014281	0.000–0.003
Propene	0.0058942	0.0048373	0.000–0.013
Acetaldehyde	0.0008888	0.0013211	0.000–0.004
Methyl Ethyl Ketone	-0.0232373	0.0353011	-0.078–0.000
M69	-0.1222845	0.1114540	-0.324–0.000
M74	-0.0266032	0.0190338	-0.052–0.000
M76	0.1684388	0.1444729	0.010–0.469
M79	-0.0489416	0.0624447	-0.146–0.000
M81	0.0425079	0.0313594	0.028–0.120
M89	-0.0280041	0.0444448	-0.127–0.000
M99	0.0793372	0.0365851	0.036–0.144
M105	-0.1427593	0.1024486	-0.271–0.000
M107	-0.0132398	0.0956782	-0.234–0.000
M115	-0.0539797	0.1593523	-0.416–0.000
M118	0.1597398	0.4747905	0.000–1.188

**Table 4:** Performance of model comparing inflammatory bowel disease patients to controls, for sensitivity levels above 90% (in parenthesis, the number of correctly classified cases).

Predicted probability	Sensitivity in overall IBD (n.67)	Specificity in overall controls (n.167)	Specificity in gastro controls (n.65)	Specificity in surgical controls (n.102)
0.0538911	100.00% (67)	37.72% (63)	34.31% (21)	41.18% (42)
0.0763976	98.51% (66)	44.31% (74)	38.46% (25)	48.04% (49)
0.1269103	97.01% (65)	59.88% (100)	56.92% (37)	61.76% (63)
0.1808475	95.52% (64)	69.46% (116)	64.62% (42)	72.55% (74)
0.1880790	92.54% (62)	70.66% (118)	66.15% (43)	73.53% (75)
0.1990687	91.04% (61)	73.05% (122)	67.69% (44)	76.47% (78)

**Table 5:** Diagnoses of the gastroenterological controls according to their classification in the model IBD vs. controls.

	Correctly classified (42)	False positives (23)	Total (65)	p*
Celiac disease	17 (40%)	5 (22%)	22 (34%)	0.173
Eosinophilic esophagitis	5 (12%)	1 (4%)	6 (9%)	0.411
Recurrent abdominal pain	1 (2%)	3 (13%)	4 (6%)	0.123
Constipation	3 (7%)	0	3 (5%)	0.547
Gastritis	1 (2%)	2 (9%)	3 (3%)	0.284
Probable latent celiac disease	1 (2%)	1 (4%)	2 (3%)	1.000
Functional dysphagia	2 (5%)	0	2 (3%)	0.536
Biliary duct atresia	2 (5%)	0	2 (3%)	0.536
Other	10 (24%)	11 (30%)	21 (32%)	0.058

* Fisher’s exact two-tailed test, considering one disease at the time vs. all others.

### CD vs. UC

The model resulting from the attempt to separate CD from UC patients is based on 13 VOCs plus age, and has the following formula (Table 6):

- predicted probability = 1/(1+exp(–(–5.178+3.444e^–01^*Age +3.465e^–03^*M29–4.342e^–02^*Acetonitrile +2.776e^–04^*NitrousOxide +4.540e^–03^*Ammonia –1.162e^–03^*Acetaldehyde +3.496e^–02^* MethylEthylKetone –6.994e^–04^*M70–1.175e^–02^*M74–2.663e^–02^*M77 +1.301e^–01^*M79–9.094e^–02^*M89–3.621e^–01^*M90–2.669e^–01^*M105–2.864e^–01^*M107 +2.873e^–01^*M114)))

**Table 6:** Variables and coefficients of the logistic regression model with outcome variables: Patients with Crohn’s disease vs. patients with ulcerative—Colitis.

Variables	Coefficient	Std. Error	95%CI
(Intercept)	-5.178	3.583	-1.065e+01 –-0.223
Age (years)	3.444e-01	3.431e-01	-4.371e-01–0.553
Ammonia	4.540e-03	5.657e-03	0.000–0.016
M29	3.465e-03	4.852e-03	0.000–0.013
Acetonitrile	-4.342e-02	5.218e-02	-1.368e-01–0.002
Nitrous Oxide	2.776e-04	7.678e-04	0.000–0.002
Acetaldehyde	-1.162e-03	2.567e-03	-7.022e-03–0.000
Methyl Ethyl Ketone	3.496e-02	3.267e-02	-3.278e-02–0.069
M70	-6.994e-04	2.479e-02	-6.398e-02–0.000
M74	-1.175e-02	3.867e-02	-9.873e-02–0.014
M77	-2.663e-02	3.456e-02	-8.980e-02–0.000
M79	1.301e-01	3.874e-01	0.000–1.049
M89	-9.094e-02	6.057e-01	-1.108–0.906
M90	-3.621e-01	1.112	-2.852–0.000
M105	-2.669e-01	6.501e-01	-1.656–0.000
M107	-2.864e-01	2.272e-01	-5.674e-01 –-0.011
M114	2.873e-01	4.202e-01	1.758e-03–1.236

The model had an AUC of 0.934 (95%CI: 0.880–0.988) and yielded a percentage of correctly classified of 86.6% (S2 Fig). It had a sensitivity of 94% in detecting CD with a specificity of 76% (Table 7). Symmetrically, the model had a sensitivity of 94% in detecting UC with a specificity of 71%.

**Table 7:** Performance of model comparing Crohn’s disease patients to ulcerative colitis patients (in parenthesis, the number of correctly classified cases).

Predicted probability	Sensitivity in Crohn Disease (n.34)	Specificity in UC controls (n.33)	Correctly classified
0.3383077	100.00% (34)	72.73% (24)	86.57%
0.3876584	94.12% (32)	75.76% (25)	85.07%
0.5131218	88.24% (30)	81.82% (27)	85.07%
0.5658435	79.41% (27)	87.88% (29)	83.58%
0.6121589	76.47% (26)	90.91% (30)	83.58%
0.6433669	70.59% (24)	93.94% (31)	82.09%
0.7598559	52.94% (18)	100.00% (33)	76.12%

### IBD vs. Gastroenterological controls

The fourth model aims at replicating a “real life” situation, in which a patient with gastrointestinal symptoms needs to be diagnosed for IBD, and is therefore designed to distinguish IBD patients from gastroenterological controls. The model is based on 15 VOCs plus age (Table 8):

- predicted probability = 1/(1+exp(–(–2.816 +4.580e^–01^*Age –1.336*Methane –3.995e^–03^ *Acetonitrile –2.463e^–01^*NitrousAcid +1.285e^–03^*AceticAcid +3.085e^–03^*Propene +8.199e^–04^ *Acetaldehyde –2.030e^–02^*M67–3.135e^–02^*M74 +7.298e^–02^*M75–8.453e^–02^*M79 +4.231e^–02^ *M81–5.543e^–02^*M89 +2.516e^–01^*M91 +5.698e^–03^*M94–1.073e^–01^*M105)))

**Table 8:** Variables and coefficients of the logistic regression model with outcome variables: Patients with inflammatory bowel disease vs. gastroenterological controls.

Variables	Coefficient	Std. Error	95% CI
(Intercept)	-2.816	4.879	-13.330–1.833
Age (years)	4.580e^-01^	2.140e^-01^	0.315–0.938
Methane	-1.336	8.425e^-01^	-2.651 –-0.107
Acetonitrile	-3.995e^-03^	1.222e^-02^	-0.032–0.000
Nitrous Acid	-2.463e^-01^	1.360e^-01^	-0.416 –-0.050
Acetic Acid	1.285e^-03^	8.897e^-04^	0.000–0.003
Propene	3.085e^-03^	4.451e^-03^	0.000–0.011
Acetaldehyde	8.199e^-04^	6.986e^-04^	0.000–0.002
M67	-2.030e^-02^	3.161e^-02^	-0.084–0.000
M74	-3.135e^-02^	2.683e^-02^	-0.073–0.000
M75	7.298e^-02^	1.361e^-01^	-0.216–0.206
M79	-8.453e^-02^	1.108e^-01^	-0.312–0.000
M81	4.231e^-02^	3.979e^-02^	0.004–0.122
M89	-5.543e^-02^	6.717e^-02^	-0.151–0.000
M91	2.516e^-01^	2.037e^-01^	0.000–0.572
M94	5.698e^-03^	8.080e^-03^	0.000–0.021
M105	-1.073e^-01^	9.137e^-02^	-0.236–0.000

The model had an AUC of 0.918 (95%CI: 0.873–0.963) (S3 Fig), and was able to identify 94% of IBDs (94% both for CD and UC patients), with a specificity of 65% (Table 9). Taking the latter as the cut-off for sensitivity and specificity, we would still have 23 false positives out of 65 gastro controls (Table 10). Once again, if the diseases are classified based on the presence or plausibility of an ongoing inflammatory process, while among the correctly classified we find one intestinal atresia, the false positives include a Behçet’s disease, a chronic intestinal pseudo-obstruction with ileostomy, a graft-versus-host disease, and an infective ileitis.

**Table 9:** Performance of model comparing patients with inflammatory bowel disease vs. gastroenterological controls, for sensitivity levels above 90% (in parenthesis, the correctly classified).

Predicted probability	Sensitivity in IBD (n.67)	Sensitivity in CD (n.34)	Sensitivity in UC (n.33)	Specificity in gastro controls (n.65)
0.1870422	100.00% (67)	100.00% (34)	100.00% (33)	46.15% (30)
0.2633038	97.01% (65)	97.06% (33)	96.97% (32)	55.38% (36)
0.2931387	95.52% (64)	94.12% (32)	96.97% (32)	58.46% (38)
0.3613650	94.03% (63)	94.12% (32)	93.94% (31)	64.62% (42)
0.3761519	92.54% (62)	94.12% (32)	90.91% (30)	66.15% (43)
0.4073664	91.04% (61)	94.12% (32)	87.88% (29)	69.23% (45)

**Table 10:** Diagnoses of the gastroenterological controls according to their classification in the model IBD vs. gastro controls.

	Correctly classified (42)	False positives (23)	Total (65)	p*
Celiac disease	14 (33%)	8 (35%)	22 (34%)	1.000
Eosinophilic esophagitis	5 (12%)	1 (4%)	6 (9%)	0.411
Recurrent abdominal pain	2 (5%)	2 (9%)	4 (14%)	0.610
Constipation	3 (7%)	0	3 (5%)	0.547
Gastritis	2 (5%)	1 (4%)	3 (5%)	1.000
Probable latent celiac disease	1 (2%)	1 (4%)	2 (3%)	1.000
Functional dysphagia	2 (5%)	0	2 (3%)	0.536
Biliary duct atresia	2 (5%)	0	2 (3%)	0.536
Other	11 (26%)	10 (43%)	21 (32%)	0.175

* Fisher’s exact two-tailed test, considering one disease at the time vs. all others.

### IBD (CD + UC) vs. controls (surgical + gastroenterological) only with directly or indirectly calibrated VOCs

Finally, the first model was replicated using only 21 unambiguously identified VOCs out of the 81 initial molecules. This version of the model was finally based on 12 VOCs plus age (Table 11):

- predicted probability = 1/(1+exp(–(–2.616+4.491e–01*Age –7.421e–01*Methane –4.356e–03*Acetonitrile +1.589e–04*NitrousOxide –3.073e–01*NitrousAcid +1.254e–03*AceticAcid +1.379e–03*Ammonia –6.212e–03*Ethylene +1.480e–03*Acetaldehyde +4.226e–04*Acetone –6.135e–03*Isopren+1.207e–01*Toluene +8.734e–03*n–Heptane)))

**Table 11:** Variables and coefficients of the logistic regression model built with directly or indirectly calibrated VOCs, with outcome variable: Patients with inflammatory bowel disease vs. controls (surgical and gastroenterological).

Variables	Estimate	Std. Error	95% CI
(Intercept)	-2.616	1.848	-4.499–0.702
Age	4.491e^-01^	8.989e^-02^	0.3.04–0.551
Methane	-7.421e^-01^	1.380	-3.952–0.000
Acetonitrile	-4.356e^-03^	4.598e^-03^	-0.012–0.000
Nitrous Oxide	1.589e^-04^	2.466e^-04^	-0.000–0.000
Nitrous Acid	-3.073e^-01^	5.877e^-02^	-0.363 –-0.208
Acetic Acid	1.254e^-03^	4.640e^-04^	0.000–0.002
Ammonia	1.379e^-03^	9.499e^-04^	0.000–0.002
Ethylene	-6.212e^-03^	9.546e^-03^	-0.025–0.002
Acetaldehyde	1.480e^-03^	9.375e^-04^	0.000–0.003
Acetone	4.226e^-04^	5.043e^-04^	0.000–0.002
Isoprene	-6.135e^-03^	2.162e^-03^	-0.009 –-0.003
Toluene	1.207e^-01^	8.372e^-02^	0.000–0.225
n-Heptane	8.734e^-03^	2.090e^-02^	0.000–0.061

As expected, the AUC of this model was smaller than the one of the model comparing IBDs with controls (AUC = 0.888; 95%CI: 0.843–0.933), but still quite high. The model was able to detect 94% of IBDs with a specificity of 61% (54% for gastro controls and 66% for surgical controls) (Table 12). If we take the latter as the cut-off for sensitivity and specificity, 30 out of 65 gastro controls result as being false positives (Table 13). Looking at the inflammatory processes, while among the correctly classified we did not find any condition to report, the false positives include a Behçet’s disease, a chronic intestinal pseudo-obstruction with ileostomy, a graft-versus-host disease, and an infective ileitis.

**Table 12:** Performance of the model with calibrated VOCs comparing patients with inflammatory bowel disease vs. controls (surgical and gastroenterological), for sensitivity levels above 90% (in parenthesis, the correctly classified).

Predicted probability	Sensitivity in overall IBD (n.67)	Specificity in overall controls (n.167)	Specificity in gastro controls (n.65)	Specificity in surgical controls (n.102)
0.0325607	100.00% (67)	24.55% (41)	23.08% (15)	25.49% (26)
0.0713997	98.51% (66)	41.92% (70)	35.38% (23)	46.08% (47)
0.0984248	97.01% (65)	48.50% (81)	41.54% (27)	52.94% (54)
0.1314432	95.52% (64)	55.09% (92)	50.77% (33)	57.84% (59)
0.1516857	94.03% (63)	61.08% (102)	53.85% (35)	65.69% (67)
0.1624737	92.54% (62)	63.47% (106)	55.38% (36)	68.63% (70)
0.1762784	91.04% (61)	65.27% (109)	56.92% (37)	70.59% (72)

**Table 13:** Diagnoses of the gastroenterological controls according to their classification in the model IBD vs. controls, built with directly or indirectly calibrated VOCs.

	Correctly classified (35)	False positives (30)	Total (65)	p*
Celiac disease	14 (40%)	8 (27%)	22 (34%)	0.301
Eosinophilic esophagitis	3 (9%)	3 (10%)	6 (9%)	1.000
Recurrent abdominal pain	1 (3%)	3 (10%)	4 (14%)	0.328
Constipation	3 (9%)	0	3 (5%)	0.241
Gastritis	1 (3%)	2 (7%)	3 (5%)	0.591
Probable latent celiac disease	2 (6%)	0	2 (3%)	0.495
Biliary duct atresia	0	2 (7%)	2 (3%)	0.209

* Fisher’s exact two-tailed test, considering one disease at the time vs. all others.

## Discussion

Among the VOCs identified by the first three models (based on 81 molecules listed in Table 2) as relevant for specific pathological conditions, five had been calibrated and quantified: Acetic Acid, Propene, Acetaldehyde, Acetonitrile and Methyl Ethyl Ketone (Table 14).

**Table 14:** Measured (the first 9 in the table) or hypothesized (the others)* VOCs that emerged as significant in our models**.

	IBD vs. Ctrls	CD vs. UC	IBD vs. Gastro Ctrls
Age (years)	+	+	+
CH_4_: Methane (MW 16)	–		–
NH_3_: Ammonia (MW 17)	+	+	
C_2_H_3_N: Acetonitrile (MW 41)		–	–
C_3_H_6_: Propene (MW 42)	+		+
N_2_O: Nitrous Oxide (MW 44)		+	
C_2_H_4_O: Acetaldehyde (MW 44)	+	–	+
HNO_2_: Nitrous Acid (47)	–		–
C_2_H_4_O_2_: Acetic Acid (MW 60)	+		+
C_4_H_8_O: Methyl Ethyl Ketone (MW 72)	–	+	
*M29*: *Methanimine (CH*_*3*_*N)*		+	
*M67*: *Pyrrole (C*_*4*_*H*_*5*_*N)*			–
*M69*: *Isocyanatoethene (C*_*3*_*H*_*3*_*NO) or 2H–imidazolium (C*_*3*_*H*_*5*_*N*_*2*_)	–		
*M70*: *Cyclopentane (C*_*5*_*H*_*10*_*) or crotonaldeyde (C*_*4*_*H*_*6*_*O)*		–	
*M74*: *Propylhidrazyne (C*_*3*_*H*_*10*_*N*_*2*_*) or allyl mercaptane (C*_*3*_*H*_*6*_*S)*	–	–	–
M75: Trimethylamine N–oxide (C_3_H_9_NO)			+
M76: Carbon disulfide (CS_2_)	+		
M77: Methyl nitrate (CH_3_NO_3_)		–	
*M79*: *Pyridine (C*_*5*_*H*_*5*_*N)*	–	+	–
*M81*: *1 or 3–Methylpyrrole (C*_*5*_*H*_*7*_*N)*	+		+
*M89*: *1–Nitropropane or 2–Nitropropane (C*_*3*_*H*_*7*_*NO*_*2*_*) or 2–(Dimethylamino)ethanol (C*_*4*_*H*_*11*_*NO)*	–	–	–
*M90*: *2*,*2–Butanediol (C*_*4*_*H*_*10*_*O*_*2*_*) or ethoxyethanol (C*_*4*_*H*_*10*_*O*_*2*_*)*		–	
*M91*: *3–Aminopropanethiol (C*_*3*_*H*_*9*_*NS)*			+
*M94*: *Phenol (C*_*6*_*H*_*6*_*O)*			+
*M99*: *Ethyl cyanoformate (C*_*4*_*H*_*5*_*NO*_*2*_*)*	+		
*M105*: *2–(Ethylamino)ethanethiol or 2–(Dimethylamino)Ethanethiol (C*_*4*_*H*_*11*_*NS)*	–	–	–
M107: 2,6–Dimethylpyridine (C_7_H_9_N)	–	–	
M114: 2,3,3–trimethylpentane (C_8_H_18_)		+	
*M115*: *1–Pyrrolidineethanol (C*_*6*_*H*_*13*_*NO) or 2–Methoxythiazole (C*_*4*_*H*_*5*_*NOS)*	–		
*M118*: *several molecules satisfy the inclusion criteria*	+		

* In italics the compounds for which we could not find evidence in the literature.

** The last three columns show the molecules retained by each model in gray; the plus or minus sign designates the sign of the coefficient in the regression model.

Acetic Acid, systematically named ethanoic acid, is commonly used in animal IBD models to reproduce an IBD condition. [42–44] Recent literature suggests that Acetic Acid and similar compounds are produced from pyruvic acid via pyruvate dehydrogenase, and that acetone is also derived from the decarboxylation of pyruvic acid. [45] It is commonly assumed that anaerobic metabolism is characterized by the non–specific production of fatty acids, such as acetic acid which is the product of several pathogens including *Staphylococcus aureus*. [46] The identification of the distinct metabolism of a specific bacteria is an important marker to determine the best pharmacological treatment.

One of the products that are derived directly from acetic acid is acetaldehyde, systematic IUPAC name ethanal, that several reports identify as a significant marker of IBD. [30,47] Acetaldehyde is present in the intestinal colon and derives from an oxidative reaction caused by several pathogens. Its antimicrobial activity in this area has been fully ascertained. [46,48] Moreover, ethanal has already been described as a potential marker for the distinction between the diagnoses of CD and UC. [49]

Propene, also known as propylene or methyl ethylene, is a hydrocarbon compound. [49] Hydrocarbon compounds are known to be products of the metabolism of gram–positive and negative bacteria. [51,52] The specific origin of propene, and consequently its role in IBDs, is unknown, but it is likely that the degradation of propene occurs through the β–oxidation pathway, as with other hydrocarbons (i.e. isoprene, 1-undecene or 1,3-butadiene).

Acetonitrile, a chemical compound also called ethanenitrile or ethyl nitrile, is mentioned in a very interesting recent report as one of nine VOCs associated with the diagnosis of esophageal adenocarcinoma: [53] there are no data linking acetonitrile to the selective diagnosis of IBDs, but this recent evidence should encourage further investigations to verify whether this compound can be considered as a suitable marker of IBD. Moreover, acetonitrile was included in an innovative study that had the objective of evaluating the VOCs profile of patients depending on their body position (sitting, standing, supine, prone, left lateral and right lateral) and cardiac output, in order to identify specific VOCs or clusters of VOCs that could be considered as biomarkers. [54]

The last compound we found is Methyl Ethyl Ketone, also known as Butanone: it has never been identified as a specific ketone in VOCs studies, but like other methyl ketones, such as acetone, is produced during decarboxylation of fatty acid derives. [55] It is worth mentioning, however, that the production of ketones through non–fermenting enterobacteriaceae has different origins. In fact, Xiao and Xu showed that acetoin, also called 3-hydroxybutadone, was detected in non-fermenting *Escherichia coli*. [56] The synthesis of acetoin in Staphylococcus has been associated with catabolic aspects of the metabolism.

Four other molecules were indirectly quantified without specific calibration: Methane, Nitrous Oxide, Nitrous Acid, and Ammonia (Table 14).

Ammonia, a well investigated inorganic compound of nitrogen and hydrogen with formula NH_3_, has been shown to be produced in greater quantities by the microbiota of IBD patients compared to healthy individuals. [57–59] A possible hypothesis to explain this result is the pivotal role of ammonia and other short–chain fatty acids in determining the onset or chronicity of IBD, since the microbiota of IBD patients synthesizes large amounts of these compounds. [50,57]

It is worth noting that published studies report lower values of ammonia in UC patients compared to controls. [29] This evidence is in contrast with that reported by other studies, [57] and helps emphasize how results can vary in populations that differ in terms of average age and number of controls and patients. [60,61]

Moreover, recently published studies have shown that ammonia is involved in protein metabolism, with the consequent production of ammonium ions that can be converted to nitric oxide in the presence of nitric oxide synthase. [62,63] NO metabolites (nitrate/nitrite) are significantly increased in IBD, and NO levels have a great potential as biomarkers for the screening of IBD. [61,64–67]

The interest on NO_X_ compounds is supported by several articles that agree in attributing to these compounds the role of biomarkers of bowel diseases. [68–70]

Finally, the production of methane in the distal colon is known to be due to endogenous (epithelial cells and dead bacteria) and exogenous (complex carbohydrates and non–digestible disaccharides) compounds. [71] Methane is an important biomarker of bacterial overgrowth typical of the IBD condition: several experimental studies tried to explain the mechanisms underlying the link between IBD and the abnormal biosynthesis of methane, [72,73] but to date this relation remains unclear. [74]

In conclusion, several VOCs show to be promising biomarkers for the non-invasive detection of IBD, thereby warranting further studies to assess whether the technical aspects of our experimental protocols on VOCs analysis can to be improved in the light of recent data in literature highlighting the importance of optimal sample collection. [75–77]

Some uncertainty remains for the other VOCs because more compounds—or fragments—may have the same MW. Following us on the hypothesis that these molecular weights refer to primary molecules, and not to fragments produced during the soft ionization and before MS detection, we compared our results with data from the literature on the composition of human alveolar air. MW 114 could correspond to 2,3,3–trimethylpentane, detected by Filipiak *et al*. [78] in the headspace of lung–cancer cells together with Acetaldehyde, MEK, Hexanal, Acrolein, and other aliphatic hydrocarbons. The same applies to MW 76, that could be identified as carbon disulphide, as detected by Navaneethan *et al*., [79] while MW 77 could be Methyl Nitrate, a product of oxidative stress reaction, as suggested by Minh *et al*. [80] MW 107 could correspond to 2,6–Dimethylpyridine, which is known to be a fragment of lysozyme [81]. MW 75 could be Trimethylamine N–oxide, a product of the microbiota, and the result of the conversion of phosphatidylcholine, a major component of cell membranes. [82–85]

For the molecules with MW 29, 67, 69, 70, 74, 79, 81, 89, 90, 91, 94, 99, 105, 115 and 118 Dalton, no data were available in literature. Thus, in order to better characterize these products, we looked at all the molecules with the above mentioned molecular weights reported by PubChem (http://pubchem.ncbi.nlm.nih.gov) or by the ChemSpider free–on–line database from the Royal Society of Chemistry (http://RSC.org; http://www.chemspider.com).

Among the reported molecules we excluded:

- Molecules of clear industrial origin (for example products containing chlorine atoms or fluorine, bromine, etc.);
- Molecules with ester linkage (as they are easily ionisable in the blood and they cannot be expelled with the alveolar air);
- Highly reactive molecules, which show instability in the biological matrix (i.e. free radicals);
- Molecules with a boiling point above 150°C, with high steam pressure (above 20 mm/Hg at 25°C) and low enthalpy of vaporization (>20 KJoule/mol): their concentration in the alveolar air should be so low that we should not be able to detect them with our equipment.

Reported in italics in Table 14 are molecules that, for their physical/chemical properties and based on the criteria identified above, could be associated with the molecular weights we identified.

We also evaluated if the performance of our models in correctly identifying CD and UC patients was affected by the level of activity of the diseases (by PCDAI and PUCAI respectively) and found no relevant relation (data not shown).

The main weakness of our study is the uncertainty in the definition of some of the molecules included in the final predictive models. Another weakness is the impossibility to recruit only CD and UC cases at onset, in the absence of an ongoing therapy that could partially affect the results of the regression models. We are aware that ideally suspect IBD cases should have been recruited at their first visit, and only subsequently divided into cases and controls, replicating a “real life” clinical situation. Selecting only suspicious cases at onset, however, would have required exceedingly long recruitment procedures. The positive aspect of our approach is that untreated cases (four CD and two UC) performed very well with all models, with predicted probabilities much higher than any possible cut–off we applied. This means that the effect of therapies, which are too complex and heterogeneous to be taken into account, is limited. The advantage of such a wide range of different therapies translates into models which are not directly affected in terms of outcome, although therapies almost certainly introduce some “noise”.

As specified in the Methods section, children had been fasting at least since midnight. Even if the prior evening meal did affect the colonic bacterial metabolism, and consequently alter the VOCs profile, we have no reason to believe meals were significantly different among the groups considered. Nevertheless, future studies might consider the possibility of standardizing the evening meal. We need to mention, however, that IBD patients might have different feeding patterns which could influence the composition of the microbiota, and consequently the VOCs pattern. In our study we did not correct for this aspect.

The main strength of the study lies in the use of a very precise instrument for the detection of VOCs. Our study clearly demonstrates that pediatric IBD patients (and CD patients in particular) have identifiable alveolar air VOCs patterns that differ from those of healthy subjects and gastroenterological controls. In addition, our models show that CD and UC present different patterns, emphasizing the different pathogenesis and clinical picture of the two diseases.

The results of the analysis of the false positives suggest that there might be something in common between IBDs and the false positives among the gastrointestinal controls, in terms of ongoing inflammatory processes. In fact, if we compare the false positives to the correctly classified in this group, we notice that the false positives have proportionally more severe and far more complex inflammatory clinical pictures. At this stage, however, this can only be a hypothesis, and certainly the intestinal microbiota, and/or the interaction between inflammation and the microbiota, could also play a role in determining the VOCs pattern.

In our opinion this study should be considered as a promising starting point. The creation of predictive models based on VOCs profiles, with the use of high precision instruments and advanced statistical methods, can contribute to the development of new non–invasive, fast and relatively inexpensive diagnostic tools, designed specifically for children, with very high sensitivity and specificity. It also represents a crucial step towards gaining further insights into the etiology of IBDs through the analysis of specific molecules which are the expression of the particular metabolism that characterizes these diseases. New prospective studies, following IBD patients from onset to post-treatment, should also be developed in order to study the relationship between VOCs profile and response to therapy.

## Supporting information

S1 Database Database including all information used in the analysis of the VOCs profiles in children with IBD vs. controls. (DTA)

S1 Fig Receiver operating characteristic curve for the model comparing IBD cases (Crohn’s disease and ulcerative colitis cases) vs. controls (both gastrointestinal and surgical controls). (PDF)

S2 Fig Receiver operating characteristic curve for the model comparing Crohn’s disease cases vs. ulcerative colitis cases. (PDF)

S3 Fig Receiver operating characteristic curve for the model comparing IBD cases (Crohn’s disease and ulcerative colitis cases) vs. Gastroenterological controls. (PDF)

S4 Fig Receiver operating characteristic curve for the model with calibrated VOCs comparing IBD cases (Crohn’s disease and ulcerative colitis cases) vs. controls (both gastrointestinal and surgical controls). (PDF)

S1 Table Mean values and 95% confidence intervals in parts per million of the 81 volatile organic compounds included in the predictive models and measured by ion-molecule reaction-mass spectrometry in environmental air and exhaled breath air samples. (PDF)

S1 Text Tables describing IBD activity indexes PUCAI and PCDAI. (PDF)

S2 Text Tables describing Crohn’s disease cases, ulcerative colitis cases and gastroenterological controls. (PDF)
