# Supplementary material for: Inflammatory bowel disease and patterns of volatile organic compounds in the exhaled breath of children: A case-control study using Ion Molecule Reaction-Mass Spectrometry
Source: PLoS One. 2017 Aug 31;12(8):e0184118. doi: 10.1371/journal.pone.0184118 (PMC5578606; doi:10.1371/journal.pone.0184118)

S2 Figure. Receiver operating characteristic curve for the model comparing Crohn's disease cases vs. ulcerative colitis cases.

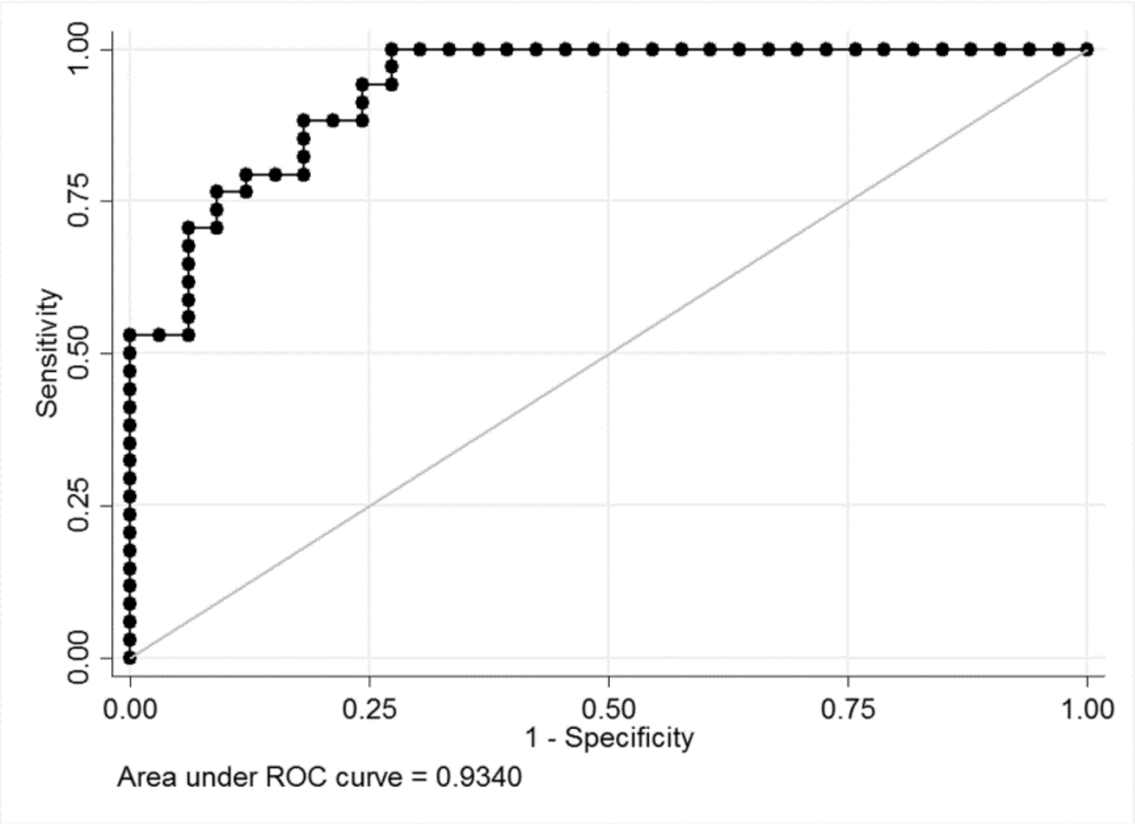

Supplement: S2 Fig — (PDF) [file pone.0184118.s003.pdf]
