# Supplementary material for: Inflammatory bowel disease and patterns of volatile organic compounds in the exhaled breath of children: A case-control study using Ion Molecule Reaction-Mass Spectrometry
Source: PLoS One. 2017 Aug 31;12(8):e0184118. doi: 10.1371/journal.pone.0184118 (PMC5578606; doi:10.1371/journal.pone.0184118)

S4 Figure. Receiver operating characteristic curve for the model with calibrated VOCs comparing IBD cases (Crohn's disease and ulcerative colitis cases) vs. controls (both gastrointestinal and surgical controls).

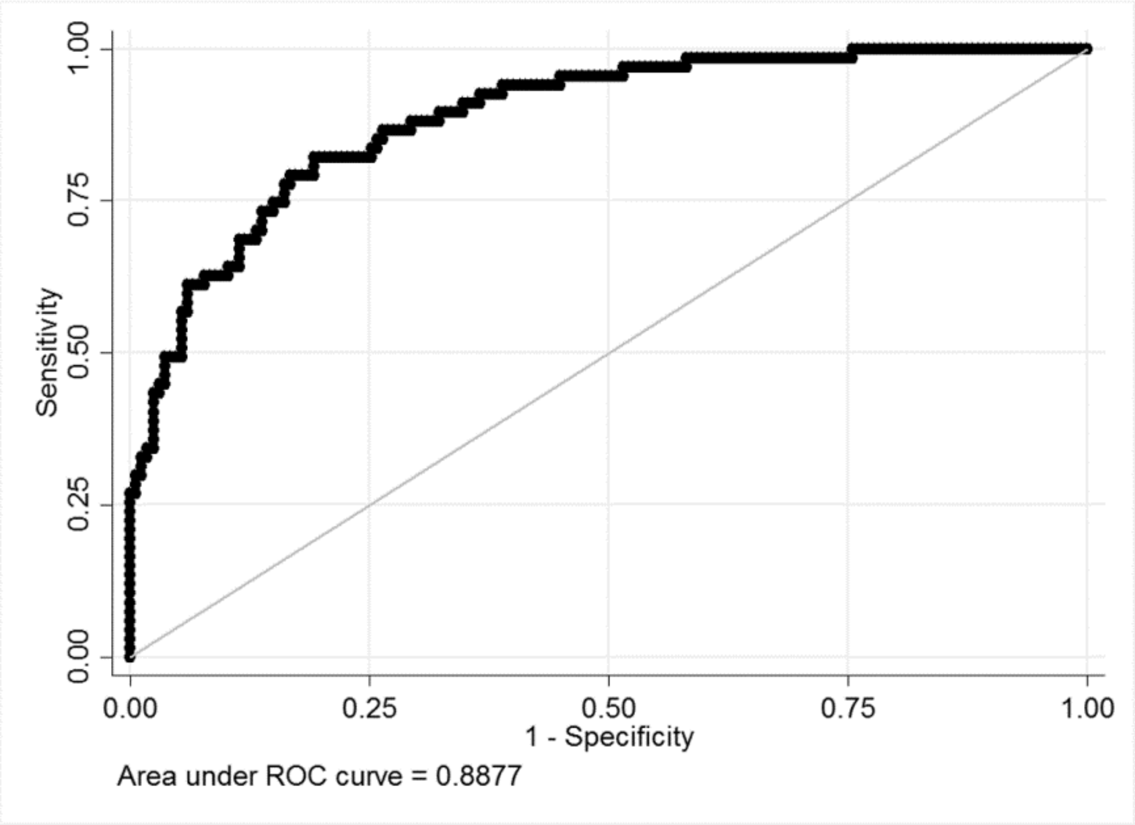

Supplement: S4 Fig — (PDF) [file pone.0184118.s005.pdf]
