# Supplementary material for: Inflammatory bowel disease and patterns of volatile organic compounds in the exhaled breath of children: A case-control study using Ion Molecule Reaction-Mass Spectrometry
Source: PLoS One. 2017 Aug 31;12(8):e0184118. doi: 10.1371/journal.pone.0184118 (PMC5578606; doi:10.1371/journal.pone.0184118)
Supplement: S1 Table — (PDF) [file pone.0184118.s006.pdf]

S1 Table. Mean values and 95% confidence intervals in parts per million of the 81 volatile organic compounds included in the predictive models, and measured by ion-molecule reaction-mass spectrometry in environmental air and exhaled breath air samples.

part 1

Molecules	Environment	Controls	Cases with Crohn's Disease	Cases with Ulcerative Colitis
CH_4 – Methane	2.023 (1.961 – 2.084)	2.241 (1.932 – 2.550)	1.434 (1.247 - 1.621)	1.454 (1.299 - 1.609)
C_2H_2 – Acetylene	55.14 (51.51 – 58.77)	57.12 (52.81- 61.43)	51.84 (40.46 - 63.22)	51.27 (41.47 - 61.06)
M29	844.5 (814.6 – 874.5)	1892 (1826 – 1958)	1883 (1734 - 2033)	1828 (1666 - 1990)
C_2H_3N – Acetonitrile	111.8 (107.6 – 115.9)	142.2 (136.8 – 147.7)	132.9 (120.7 - 145.1)	135 (123.9 – 146.0)
N_2O – Nitrous Oxide	321.3 (307.8 – 334.7)	4007 (3770 – 4245)	4414 (3773 - 5056)	3969 (3389 - 4548)
HNO_2 – Nitrous Acid	7.537 (5.736 – 9.337)	21.57 (20.52 – 22.61)	18.84 (16.43 - 21.24)	17.89 (15.86 - 19.92)
$C_2H_4O_2$ – Acetic Acid	184.8 (178.9 – 190.7)	772.8 (712.1 – 833.5)	2285 (149 - 4421)	1409 (575 - 2244)
NH_3 – Ammonia	184.2 (170.7 – 197.7)	352.4 (323.8 – 381.1)	387.5 (311.1 - 464)	368 (304 - 431.9)
M19	214.5 (200.9 – 228.1)	936.9 (830.6 – 1043.1)	873.9 (618.2 - 1129.7)	884.8 (585.8 - 1183.8)
C_2H_4 – Ethylene	169.7 (161.6 – 177.8)	173.0 (162.8 – 183.2)	157.1 (133.4 - 180.9)	160.3 (135.8 - 184.8)
M33	8.198 (7.739 – 8.657)	30.51 (27.23 – 33.80)	48.28 (26.09 - 70.47)	58.13 (21.92 - 94.34)
C_3H_6 – Propene	160.2 (151.9 – 168.5)	277.3 (260.8 – 293.8)	345.7 (280 - 411.4)	354.1 (260.4 - 447.7)
M43	304.9 (294.8 – 315.1)	901.5 (819.7 – 983.2)	1468 (981 - 1956)	1606 (783 - 2429)
C_2H_4O – Acetaldehyde	181.8 (150.7 – 212.9)	616.0 (546.9 – 685.1)	762.5 (581.4 - 943.6)	843.6 (615.6 - 1071.5)
M45	372.6 (268.6 – 476.6)	295.0 (266.3 – 323.6)	298.2 (226.1 - 370.3)	341 (212.7 - 469.3)
C_4H_6 – Butadiene	8.755 (8.445 – 9.066)	11.82 (11.42 – 12.23)	11.31 (9.99 - 12.64)	11.52 (10.69 - 12.34)
$C_4H_{10}O$ – Butanol	130.0 (124.5 – 135.5)	173.5 (166.8 – 180.1)	171.6 (151.6 - 191.5)	173.6 (156.7 - 190.6)
C_4H_8O – Methyl Ethyl Ketone	40.67 (39.18 – 42.17)	59.78 (56.81 – 62.75)	72.2 (59.73 - 84.68)	65.75 (53.77 - 77.72)
C_3H_6O – Acetone	110.0 (103.9 – 116.1)	584.0 (518.5 – 649.4)	1047 (611 - 1483)	1114 (450 - 1778)
C_3H_8O – n-Propanol	38.53 (33.65 – 43.40)	109.6 (101.7 – 117.4)	162.8 (119.4 - 206.2)	186.4 (88.3 - 284.5)
M60	9.492 (8.897 – 10.099)	39.82 (37.40 – 42.24)	52.35 (38.63 - 66.07)	53.93 (34.84 - 73.01)
M61	3.644 (3.412 – 3.877)	85.73 (75.32 – 96.13)	140 (100.2 - 179.7)	170.5 (60.8 - 280.3)
M62	1.414 (1.287 – 1.542)	10.18 (9.36 – 11.00)	11.38 (9.35 - 13.42)	12.24 (7.07 - 17.4)
M63	2.954 (2.453 – 3.456)	8.364 (7.728 – 8.999)	9.522 (7.754 - 11.29)	10.94 (5.96 - 15.92)
M66	3.220 (3.008 – 3.432)	5.782 (5.376 – 6.189)	6.122 (5.3 - 6.944)	6.026 (5.316 - 6.736)
M67	7.728 (7.338 – 8.118)	25.47 (23.66 – 27.29)	24.63 (21.53 - 27.73)	25.54 (21.1 - 29.98)
C_5H_8 – Isoprene	32.57 (31.37 – 33.77)	173.7 (161.3 – 186.1)	158.4 (140.8 - 176.1)	165.3 (137.2 - 193.3)
M69	34.86 (33.74 – 35.98)	43.44 (42.25 – 44.64)	40.38 (37.16 - 43.6)	42.49 (39.64 - 45.34)
M70	65.75 (60.46 – 71.03)	80.09 (77.61 – 82.58)	74.94 (68.44 - 81.44)	86.53 (67.4 - 105.66)
M71	24.61 (23.02 – 26.20)	36.40 (35.06 – 37.75)	37.74 (34.05 - 41.44)	40.68 (34.58 - 46.79)
C_5H_{12} – n-Pentane	29.70 (28.77 – 30.64)	58.43 (54.77 – 62.09)	72.14 (56.49 - 87.79)	70.47 (48.94 - 92)
M73	5.281 (4.522 – 6.040)	17.06 (15.96 – 18.17)	19.26 (14.81 - 23.71)	20.92 (14.38 - 27.45)
M74	5.665 (5.305 – 6.024)	33.59 (29.94 – 37.24)	28.91 (22.44 - 35.38)	32.34 (25.15 - 39.52)
M75	1.393 (1.260 – 1.527)	6.997 (6.598 – 7.396)	7.473 (6.319 - 8.628)	8.278 (6.101 - 10.455)
M76	1.204 (1.087 – 1.322)	6.729 (6.207 – 7.251)	8.586 (6.627 - 10.545)	10.56 (5.55 - 15.57)
M77	6.363 (6.090 – 6.636)	31.94 (28.43 – 35.44)	35.51 (22.4 - 48.62)	54.51 (18.99 - 90.04)
C_6H_6 – Benzene	8.934 (8.478 – 9.389)	68.25 (64.50 – 72.00)	62.58 (51.83 - 73.33)	61.33 (52.78 - 69.89)
M79	3.970 (3.584 – 4.357)	9.022 (8.284 – 9.761)	10.31 (8.06 - 12.55)	10.18 (7.91 - 12.46)
M81	45.09 (43.14 – 47.04)	63.50 (60.99 – 66.01)	63.98 (57.95 - 70.01)	80.29 (50.56 - 110.03)
M82	69.17 (66.46 – 71.88)	97.92 (94.29 – 101.54)	98.39 (90.52 - 106.26)	103.9 (94.9 - 112.9)
M83	93.08 (89.24 – 96.91)	123.3 (118.8 – 127.7)	126.6 (112.8 - 140.5)	130.5 (116.8 - 144.3)
M84	135.2 (129.9 – 140.5)	185.5 (178.3 – 192.6)	196.9 (171.3 - 222.6)	201 (174.2 - 227.9)
M85	118.3 (112.2 – 124.4)	144.1 (136.7 – 151.5)	152.3 (128.5 - 176.1)	148.7 (130.7 - 166.7)
C_6H_{14} – n-Hexane	186.6 (175.8 – 197.4)	214.9 (201.6 – 228.3)	243.4 (191.2 - 295.7)	219.8 (186.3 - 253.3)
M87	21.53 (20.55 – 22.51)	49.02 (47.01 – 51.02)	52.38 (43.21 - 61.55)	54.77 (41.01 - 68.53)
M88	9.381 (8.912 – 9.850)	41.12 (38.54 – 43.70)	41.01 (32.41 - 49.62)	45.29 (31.85 - 58.73)
M89	1.453 (1.172 – 1.734)	14.37 (12.66 – 16.09)	12.34 (9.83 - 14.85)	14.7 (9.45 - 19.94)

part 2

Molecules	Environment	Controls	Cases with Crohn's Disease	Cases with Ulcerative Colitis
M90	1.083 (.952 – 1.214)	3.815 (3.589 – 4.041)	3.693 (3.128 - 4.257)	4.049 (3.286 - 4.812)
M91	3.219 (2.902 – 3.535)	5.626 (5.288 – 5.964)	6.087 (5.095 - 7.079)	6.894 (5.653 - 8.136)
C_7H_8 – Toluene	4.645 (4.304 – 4.985)	8.629 (8.249 – 9.008)	8.631 (7.584 - 9.679)	9.493 (8.051 - 10.935)
M93	27.15 (25.59 – 28.70)	65.49 (62.26 – 68.73)	59.8 (51.41 - 68.2)	64.85 (57.14 - 72.56)
M94	54.50 (51.70 – 57.30)	153.6 (145.4 – 161.7)	148.4 (124.8 - 172.1)	180.7 (133 - 228.4)
M95	44.75 (42.97 – 46.53)	124.2 (115.1 – 133.4)	133 (94.4 - 171.5)	186.3 (84.5 - 288)
M96	64.77 (61.92 – 67.62)	99.00 (95.40 – 102.59)	98.19 (88.7 - 107.67)	107.1 (94.9 - 119.2)
M97	73.25 (65.49 – 81.02)	82.33 (77.44 – 87.23)	86.85 (74.99 - 98.71)	91.47 (78.7 - 104.25)
M98	101.7 (86.8 – 116.6)	95.10 (87.12 – 103.08)	97.77 (77.97 - 117.57)	104.6 (83.3 - 125.9)
M99	48.10 (44.90 – 51.29)	62.89 (60.79 – 64.99)	66.34 (60.51 - 72.16)	70.3 (60.84 - 79.76)
C_7H_{16} – n-Heptane	36.15 (31.44 – 40.87)	66.30 (64.27 – 68.33)	62.58 (57.04 - 68.12)	75.52 (55.86 - 95.18)
M101	4.007 (3.6517 – 4.363)	15.75 (14.80 – 16.69)	18.12 (14.86 - 21.39)	22.73 (13.18 - 32.28)
M102	2.410 (2.260 – 2.559)	15.35 (14.12 – 16.58)	18.35 (13.81 - 22.89)	24.18 (12 - 36.37)
M103	2.682 (2.466 – 2.899)	16.41 (14.54 – 18.29)	20.55 (13.29 - 27.82)	28.95 (11.72 - 46.17)
M104	3.650 (3.390 – 3.909)	10.44 (9.77 – 11.10)	11.03 (8.88 - 13.17)	13.34 (9.22 - 17.45)
M105	6.299 (5.972 – 6.627)	12.63 (12.03 – 13.23)	12.47 (11.06 - 13.88)	13.51 (12.13 - 14.89)
M106	7.807 (7.449 – 8.165)	13.78 (13.15 – 14.42)	13.82 (12.43 - 15.22)	14.48 (12.86 - 16.1)
M107	12.53 (11.97 – 13.09)	16.73 (16.06 – 17.40)	14.79 (13.19 - 16.39)	17.15 (14.66 - 19.65)
M108	26.16 (25.00 – 27.32)	31.77 (30.41 – 33.14)	29.5 (25.85 - 33.14)	33.53 (28.22 - 38.85)
M109	22.18 (21.19 – 23.17)	28.29 (27.00 – 29.58)	31.02 (27.05 - 35)	30.88 (27.44 - 34.33)
M110	22.59 (21.37 – 23.80)	34.35 (32.75 – 35.95)	36.81 (32.93 - 40.69)	37.36 (34.17 - 40.56)
M111	16.71 (15.81 – 17.62)	27.64 (26.49 – 28.79)	30.35 (27.35 - 33.35)	29.98 (27.16 - 32.79)
M112	19.05 (18.01 – 20.08)	38.83 (37.09 – 40.56)	43.02 (35.52 - 50.52)	42.46 (35.23 - 49.68)
M113	9.873 (9.230 – 10.516)	28.30 (26.50 – 30.09)	31.89 (23.49 - 40.28)	35.03 (20.14 - 49.92)
M114	8.805 (8.412 – 9.197)	20.65 (19.84 – 21.46)	21.75 (18.83 - 24.67)	21.34 (17.4 - 25.28)
M115	1.523 (1.370 – 1.677)	10.59 (10.04 – 11.13)	12.19 (9.42 - 14.95)	12.1 (7.59 - 16.61)
M116	.8066 (.7324 – .8808)	6.285 (5.936 – 6.634)	6.595 (5.107 - 8.083)	7.177 (4.751 - 9.603)
M117	.7256 (.6542 – .7971)	5.544 (5.119 – 5.968)	5.865 (4.531 - 7.199)	5.773 (4.318 - 7.228)
M118	1.223 (1.128 – 1.317)	3.130 (2.956 – 3.304)	3.205 (2.739 - 3.67)	3.409 (2.908 - 3.909)
M119	2.312 (2.166 – 2.457)	2.918 (2.750 – 3.086)	3.276 (2.778 - 3.774)	3.2 (2.837 - 3.563)
M120	3.514 (3.260 – 3.768)	4.066 (3.802 – 4.331)	4.793 (3.934 - 5.653)	4.683 (4.069 - 5.296)
M121	3.405 (3.160 – 3.650)	5.416 (5.079 – 5.754)	5.966 (5.155 - 6.777)	5.972 (5.305 - 6.638)
M122	8.727 (8.319 – 9.135)	11.83 (11.26 – 12.41)	12.71 (11.28 - 14.14)	13.46 (11.88 - 15.04)
M123	19.66 (18.83 – 20.48)	22.13 (21.27 – 22.99)	23.61 (21.19 - 26.02)	24.91 (22.09 - 27.72)
