# Supplementary material for: Inflammatory bowel disease and patterns of volatile organic compounds in the exhaled breath of children: A case-control study using Ion Molecule Reaction-Mass Spectrometry
Source: PLoS One. 2017 Aug 31;12(8):e0184118. doi: 10.1371/journal.pone.0184118 (PMC5578606; doi:10.1371/journal.pone.0184118)
Supplement: S1 Text — (PDF) [file pone.0184118.s007.pdf]

S1A Table. Pediatric Ulcerative Colitis Activity Index (PUCAI)

Item	Points
**1. Abdominal pain**:	
No pain	0
Pain can be ignored	5
Pain cannot be ignored	10
**2. Rectal bleeding**	
None	0
Small amount only, in less than 50% of stools	10
Small amount with most stools	20
Large amount (>50% of the stool content)	30
**3. Stool consistency of most stools**	
Formed	0
Partially formed	5
Completely unformed	10
**4. Number of stools per 24 hours**	
0-2	0
3-5	5
6-8	10
>8	15
**5. Nocturnal stools (any episode causing wakening)**	
No	0
Yes	10
**6. Activity level**	
No limitation of activity	0
Occasional limitation of activity	5
Severe restricted activity	10
**SUM OF PUCAI (0-85)**	

© Copyright to The Hospital for Sick Children, Toronto, Canada, 2006.

From: Turner D, Otley AR, Mack D, et al. Development, validation, and evaluation of a pediatric ulcerative colitis activity index: a prospective multicenter study. Gastroenterology. 2007;133(2):423-32.

S1B Table. Pediatric Crohn's Disease Activity Index (PCDAI)

**HISTORY (Recall: 1 week)**		
**Abdominal pain**		
None		0
Mild-- Brief, does not interfere with activities		5
Mod/severe - daily, longer lasting affects activities, nocturnal		10
**Stools (per day)**		
Formed stools or up to 1 liquid stool, no blood		0
Up to 2 semi-formed with small blood, or 2-5 liquid with or without small blood		5
Any gross bleeding, or ≥ 6 liquid, or nocturnal diarrhea		10
**Patient Functioning -- General Well-Being**		
No Limitation of activities, well		0
Occasional difficulty in maintaining appropriate activities, below par		5
Frequent limitation of activity, very poor		10
**EXAMINATION**		
**Weight**		
Weight gain or voluntary weight stable/loss		0
Involuntary weight stable, weight loss 1-9%		5
Weight loss ≥ 10%		10
**Height**		
**At diagnosis**	<1 channel decrease	0
	≥1,<2 channel decrease	5
	>2 channel decrease	10
**Follow-up**	Height velocity ≥ —1SD	0
	Height velocity < —1SD, > —2SD	5
	Height velocity ≤ —2SD	10
**Abdomen**		
No tenderness, no mass		0
Tenderness, or mass without tenderness		5
Tenderness, involuntary guarding, definite mass		10
**Perirectal disease**		
None, asymptomatic tags		0
Inflamed tags or 1-2 indolent fistula(e) or fissure(s), scant drainage, no tenderness		5
Active fistula, drainage, tenderness, or abscess		10
**Extra-intestinal Manifestations**		
(Fever ≥ 38.5°C for 3 days over past week, oral ulcers, definite arthritis, uveitis, erythema nodosum, pyoderma gangrenosum)	None	0
	One	5
	≥ Two	10

LABORATORY					
**HCT (%)**	**<10 years**	**11-19 yrs F**	**11-14 yrs M**	**15-19 yrs M**	
	>33	>34	>35	>37	0
	28-32	29-33	30-34	32-36	2.5
	<28	<29	<30	<32	5
**ESR (mm/hr)**					
	<20				0
	20-50				2.5
	>50				5
**Albumin (gr/dL)**					
	>3.5				0
	3.1-3.4				2.5
	<3.0				5
**TOTAL SCORE (0-105)**					

From: Hyams JS, Ferry GD, Mandel FS, et al. Development and validation of a pediatric Crohn's disease activity index. J Pediatr Gastroenterol Nutr. 1991;12:439-447.

S1C Table. Description of the Crohn's disease cases enrolled in the study (n=34)

	Frequencies (percentages) or Median (IQR)
Age at onset:	
- A1a (0 to 9 years)	11 (32%)
- A1b (10 to 17 years)	23 (68%)
Location:	
- L1, terminal ileum	7 (21%)
- L2, colon	4 (12%)
- L3, ileocolon	12 (35%)
- L4, upper gastrointestinal	2 (6%)
- L3 + L4	6 (18%)
- L3 + L5	1 (3%)
- L3 + L4 + L5	2 (6%)
Behavior:	
- B1, non-stricturing non-penetrating	18 (53%)
- B2, stricturing	13 (38%)
- B3, penetrating	2 (6%)
-B2 + B3	1 (3%)
Growth delay:	
- G0, no evidence of growth delay	12 (35%)
- G1, growth delay	22 (65%)
PCDAI=0	11 (32%)
PCDAI if PCDAI≠0 (n=23)	10.0 (5.0 - 17.5)
Perianal disease, yes	5/34 (15%)
Associated pathology	
- No	24 (71%)
- Dermatologic diseases	6 (18)
- Hepatic diseases	1 (3%)
- Eye diseases	1 (3%)
- Oligoarticular arthritis	1 (3%)
- Eosinophilic Enterocolitis	1 (3%)
Therapies:	
- Azathioprine	9
- Thalidomide	8
- Infliximab/Adaliumab	4
- Cortisone/corticosteroids + Azathioprine	3
- Antibiotics	2
- Methotrexate	2
- Other therapies	5
- No therapy	1

IQR: interquartile range.

S1D Table. Description of the Ulcerative Colitis cases enrolled in the study (n=33)

	Frequencies (percentages) or Median (IQR)
Extent:	
- E1, Proctitis	1 (3%)
- E2, Left sided	6 (18%)
- E3, Extensive	3 (9%)
- E4, Pancolitis	23 (70%)
Severity:	
- S0, never severe	26 (79%)
- S1, ever severe	7 (21%)
PUCAI=0	19 (58%)
PUCAI if PUCAI≠0 (n=14)	22.5 (10 - 45)
Associated pathology	
- No	24 (73%)
- Hepatic diseases	7 (21%)
- Previous juvenile idiopathic arthritis	1 (3%)
- Idiopathic portal hypertension	1 (3%)
Therapies:	
- Mesalazine/Sulfasalazine	11
- Azathioprine + Mesalazine/Sulfasalazine	10
- Mesalazine/Sulfasalazine + Thalidomide	2
- Mesalazine/Sulfasalazine + Infliximab/Adaliumab	2
- Other therapies	6
- No therapy	2

S1E Table. Diagnoses of the Gastroenterological controls enrolled in the study (n=65)

**Diagnoses**	**Frequencies (percentages)**
Celiac disease	22 (34%)
Eosinophilic esophagitis	6 (9%)
Recurrent abdominal pain	4 (6%)
Constipation	3 (5%)
Gastritis	3 (5%)
Probable latent celiac disease	2 (3%)
Functional dysphagia	2 (3%)
Biliary duct atresia	2 (3%)
Other (all single frequencies)	21 (32%)
