# Supplementary material for: Inflammatory bowel disease and patterns of volatile organic compounds in the exhaled breath of children: A case-control study using Ion Molecule Reaction-Mass Spectrometry
Source: PLoS One. 2017 Aug 31;12(8):e0184118. doi: 10.1371/journal.pone.0184118 (PMC5578606; doi:10.1371/journal.pone.0184118)
Supplement: S2 Text — (PDF) [file pone.0184118.s008.pdf]

S2A Table. Description of the Crohn's disease cases enrolled in the study (n=34)

	Frequencies (percentages) or Median (IQR)
Age at onset:	
- A1a (0 to 9 years)	11 (32%)
- A1b (10 to 17 years)	23 (68%)
Location:	
- L1, terminal ileum	7 (21%)
- L2, colon	4 (12%)
- L3, ileocolon	12 (35%)
- L4, upper gastrointestinal	2 (6%)
- L3 + L4	6 (18%)
- L3 + L5	1 (3%)
- L3 + L4 + L5	2 (6%)
Behavior:	
- B1, non-stricturing non-penetrating	18 (53%)
- B2, stricturing	13 (38%)
- B3, penetrating	2 (6%)
-B2 + B3	1 (3%)
Growth delay:	
- G0, no evidence of growth delay	12 (35%)
- G1, growth delay	22 (65%)
PCDAI=0	11 (32%)
PCDAI if PCDAI≠0 (n=23)	10.0 (5.0 - 17.5)
Perianal disease, yes	5/34 (15%)
Associated pathology	
- No	24 (71%)
- Dermatologic diseases	6 (18)
- Hepatic diseases	1 (3%)
- Eye diseases	1 (3%)
- Oligoarticular arthritis	1 (3%)
- Eosinophilic Enterocolitis	1 (3%)
Therapies:	
- Azathioprine	9
- Thalidomide	8
- Infliximab/Adaliumab	4
- Cortisone/corticosteroids + Azathioprine	3
- Antibiotics	2
- Methotrexate	2
- Other therapies	5
- No therapy	1

IQR: interquartile range.

S2B Table. Description of the Ulcerative Colitis cases enrolled in the study (n=33)

	Frequencies (percentages) or Median (IQR)
Extent:	
- E1, Proctitis	1 (3%)
- E2, Left sided	6 (18%)
- E3, Extensive	3 (9%)
- E4, Pancolitis	23 (70%)
Severity:	
- S0, never severe	26 (79%)
- S1, ever severe	7 (21%)
PUCAI=0	19 (58%)
PUCAI if PUCAI≠0 (n=14)	22.5 (10 - 45)
Associated pathology	
- No	24 (73%)
- Hepatic diseases	7 (21%)
- Previous juvenile idiopathic arthritis	1 (3%)
- Idiopathic portal hypertension	1 (3%)
Therapies:	
- Mesalazine/Sulfasalazine	11
- Azathioprine + Mesalazine/Sulfasalazine	10
- Mesalazine/Sulfasalazine + Thalidomide	2
- Mesalazine/Sulfasalazine + Infliximab/Adaliumab	2
- Other therapies	6
- No therapy	2

S2C Table. Diagnoses of the Gastroenterological controls enrolled in the study (n=65)

**Diagnoses**	**Frequencies (percentages)**
Celiac disease	22 (34%)
Eosinophilic esophagitis	6 (9%)
Recurrent abdominal pain	4 (6%)
Constipation	3 (5%)
Gastritis	3 (5%)
Probable latent celiac disease	2 (3%)
Functional dysphagia	2 (3%)
Biliary duct atresia	2 (3%)
Other (all single frequencies)	21 (32%)
